# Effect of mental fatigue on hand force production capacities

**DOI:** 10.1371/journal.pone.0298958

**Published:** 2024-04-02

**Authors:** Thomas Jacquet, Bénédicte Poulin-Charronnat, Patrick Bard, Romuald Lepers

**Affiliations:** 1 Faculté des Sciences du Sport, CAPS, Inserm U1093, BP 27877 UFR STAPS, Université de Bourgogne, Dijon, France; 2 LEAD – CNRS UMR5022, Université de Bourgogne, Dijon, France; Università degli Studi di Milano: Universita degli Studi di Milano, ITALY

## Abstract

Mental fatigue is common in society, but its effects on force production capacities remain unclear. This study aimed to investigate the impact of mental fatigue on maximal force production, rate of force development-scaling factor (RFD-SF), and force steadiness during handgrip contractions. Fourteen participants performed two randomized sessions, during which they either carried out a cognitively demanding task (i.e., a visual attention task) or a cognitively nondemanding task (i.e., documentary watching for 62 min). The mental fatigue was evaluated subjectively and objectively (performances and electroencephalography). Maximal voluntary contraction (MVC) force, RFD-SF, and force steadiness (i.e., force coefficient of variation at submaximal intensities; 25, 50, and 75% of MVC) were recorded before and after both tasks. The feeling of mental fatigue was much higher after completing the cognitively demanding task than after documentary watching (*p* < .001). During the cognitively demanding task, mental fatigue was evidenced by increased errors, missed trials, and decreased N100 amplitude over time. While no effect was reported on force steadiness, both tasks induced a decrease in MVC (*p* = .040), a force RFD-SF lower slope (*p* = .011), and a reduction in the coefficient of determination (*p* = .011). Nevertheless, these effects were not explicitly linked to mental fatigue since they appeared both after the mentally fatiguing task and after watching the documentary. The study highlights the importance of considering cognitive engagement and mental load when optimizing motor performance to mitigate adverse effects and improve force production capacities.

## Introduction

Mental fatigue is a psychobiological state caused by prolonged and/or intense periods of cognitively demanding activity and characterized by subjective feelings of “tiredness” and “lack of energy” [1] that can impair sport performance [for review: 2, 3]. The effects of mental fatigue on sporting performance are intricately linked to alterations in both cognitive and physical capacities. It is now well established that mental fatigue impairs attention [4] or inhibition capacities [5], but also sport-related decision-making, technical skills, motor control, and endurance performances [for review: 5]. However, the effects of mental fatigue on force production capacities remained unclear.

In studies looking at mental fatigue, force production capacities have been mainly investigated throughout maximal force production. While recent reviews generally suggest that mental fatigue does not significantly impact maximal force production [1, 3], a growing number of studies revealed a decline in maximal voluntary contraction (MVC) following mentally fatiguing tasks, during knee extension [6] or dorsiflexion contractions [7]. Even if the maximal voluntary force is a major factor in the force production capacities, the rate of force development (RFD) appeared to be more related to most performance in sport-specific and functional daily tasks [8] and more sensitive for detecting neuromuscular changes [9]. RFD represents the ability to develop force as quickly as possible during a rapid MVC. A linear increase in RFD was observed at rising force amplitudes: the higher the force required, the quicker the contraction [10].

In everyday life and sports practice, most activities are performed at submaximal intensities. For this reason, the RFD measured during contraction at submaximal intensities could have higher ecological validity than RFD measured during maximal contractions [11]. In this context, protocols using the RFD scaling factor (RFD-SF) provide an appealing approach [12]. RFD-SF mainly reflects the neural mechanisms of RFD (i.e., motor unit firing rate) and is independent of other RFD determinants, such as maximal strength or muscle fiber type [13]. Although physical fatigue does not seem to affect the RFD-SF [11], this latter could be impacted positively by resistance exercise [14] or negatively by aging [15]. To date, studies on the effects of mental fatigue on RFD have been scarce. Although no effect of mental fatigue has been demonstrated [16], peripherical changes have been observed in the presence of mental fatigue with a decrease in electromyographic (EMG) activity during submaximal knee extensions [17] or cycling exercise at 80% of peak power output [18], associated with slower motor unit firing rate at submaximal intensities [19]. The decrease in motor unit firing rate at submaximal intensities could alter RFD-SF in the presence of mental fatigue. Electromyography (EMG) activity is a significant factor that affects force steadiness, which is the variability of force production at a given target force [20]. Yao et al. [21] showed that an increase in EMG amplitude is associated with a decrease in force steadiness. Several factors, such as muscle fatigue [22] or aging [23], have been studied for their effects on force steadiness, but limited research has been done on the effects of mental fatigue on this parameter. Although Budini et al. [24] did not observe significant changes in force steadiness (quantified in terms of standard deviation and coefficient of variation) during pinch contractions, other studies have indicated that engaging in cognitively demanding tasks can lead to enhanced force steadiness evaluated by the coefficient of variation of force [25] or by the root mean square of the force signal [26].

In studies investigating the effects of mental fatigue on sports performance, particularly on force production capacities, the assessment of mental fatigue has mainly relied on subjective scales. While the visual analogue scale has been reported by Smith et al. [27] as the most practical method for assessing mental fatigue, the use of electrophysiological measures seems important to objectively evidence its presence. Electroencephalography (EEG) is now commonly used to measure mental fatigue objectively. Consistent findings reveal changes in brain oscillations, specifically an increase in theta and alpha power, as robust markers of mental fatigue [for review: 28]. By utilizing EEG, researchers can objectively evidence the presence of mental fatigue before exploring its potential impact on maximal force production capacities. In this context, the present study aimed to investigate the effects of mental fatigue on force production capacities, with a particular focus on maximal force production, rate of force development scaling factor (RFD-SF), and force steadiness during handgrip contractions. We decided to focus on hand contractions, as they correspond to many manual tasks in everyday life or sporting movements (e.g., racket sports). We hypothesized that hand strength capacities would be more altered following a high-cognitive demanding task than following a nondemanding cognitive demanding task, and would be associated with greater changes in brain oscillations.

## Materials and methods

### Participants

Fourteen physically active students of the University of Bourgogne (seven female) with normal or corrected-to-normal vision and no history of neurological disease volunteered for this study (mean ± SD; age: 22.6 ± 2.3 years, height: 168.6 ± 5.8 cm, weight: 67.8 ± 3.6 kg). The recruitment period for the study was from January 1 2021 to June 30, 2021. To check that they were all right-handed, participants completed the Edinburg questionnaire. All participants were instructed to sleep for at least six hours, not consume alcohol, and not practice vigorous physical activity the day before each visit. They were also advised not to consume caffeine or nicotine for at least three hours before testing. They were asked to declare if they had taken any medication or had any acute illness, injury, or infection. The experiment was conducted in conformity with the latest version of the Declaration of Helsinki (1964) and approved by the local Ethics Committee of the Université Bourgogne Franche-Comté (CERUBFC-2021-05-12-010). All participants provided written informed consent.

### Experimental tasks

#### Cognitively demanding task

The cognitively demanding task is a visual attention task already used successfully to induce mental fatigue [4]. Each experimental block began with the presentation of a fixation cross, which remained on screen throughout the entire block. Then, a memory set of two letters was displayed on a screen for 2000 ms. Stimulus letters were randomly chosen from the alphabet, excluding the letters G, I, O, Q, U, X, and Y. Next, two black squares (left-up or right-up diagonal) were presented to indicate which display positions were relevant. A series of 160 stimulus displays (constituting one block) were pseudorandomly presented to participants during 50 ms with a randomized interstimulus interval between 1000 and 1500 ms. The stimulus display contained two letters randomly shown on either the left-up (50%) or the right-up (50%) diagonal positions. The memory set items appeared at a relevant diagonal position (relevant target) in 25% of the trials and at an irrelevant diagonal position (irrelevant target) in 25% of other trials. Trials without memory-set items appeared in 25% of trials at the relevant diagonal position (relevant nontarget) and 25% of other trials at the irrelevant diagonal position (irrelevant nontarget). During the experimental session, participants performed 50 blocks of 160 trials for 62 min in total. Participants had to press the "f" key on the keyboard with their left index finger when the memory-set items appeared at a relevant diagonal position, and the "j" key with their right index finger for all other trials. When the participant pressed the wrong button, it was considered an error; when she/he did not press any button, it was considered a missed trial.

#### Documentary watching

Participants watched an emotionally neutral documentary, “Home” by Y. Arthus-Bertrand, which was used as a control condition in previous studies on mental fatigue [16]. Watching a movie has been recently evidenced as a suitable control condition in studies about mental fatigue [29]. Participants were sitting in a dark room at the same as for the cognitively demanding task, and the movie lasted 62 min, the same duration as for the cognitively demanding task.

#### Handgrip task

The handgrip task consisted of performing three MVCs, followed by 30 submaximal contractions at 25, 50, and 75% of MVC, and three MVCs at the end of the exercise. For MVC, participants were asked to clasp the handgrip as hard as possible for 3 s with an instructor’s verbal encouragement. For submaximal contractions, participants were instructed to reach the target force “as fast and explosive as possible” [9]. A delay of 60 s was respected between each MVC with at least 6 s between each submaximal contraction. A visual stimulus appeared on a screen in front of the participants to inform them that they could perform the contraction. Participants were free to start the contractions when they wanted after the visual stimulus apparition. Afterward, participants performed three prolonged isometric contractions in random order at 25, 50, and 75% of the MVC. The contractions lasted 15 s, with an audio signal to start and stop the contractions and 30 s between each contraction. Visual feedback of the force developed was presented on a screen in front of the participants to match the target force level required as accurately as possible.

### Procedure

Participants visited the laboratory on three different occasions. Each participant completed all three visits over three weeks with a minimum of 48 h of recovery between visits. All experiments started between 8:00 a.m. and 10:00 a.m. The first visit aimed to familiarize the participants with all the procedures. All questionnaires were presented and explained to the participants during the following sessions. Then, participants performed the handgrip task, followed by four blocks of the cognitively demanding task.

**Fig 1:**
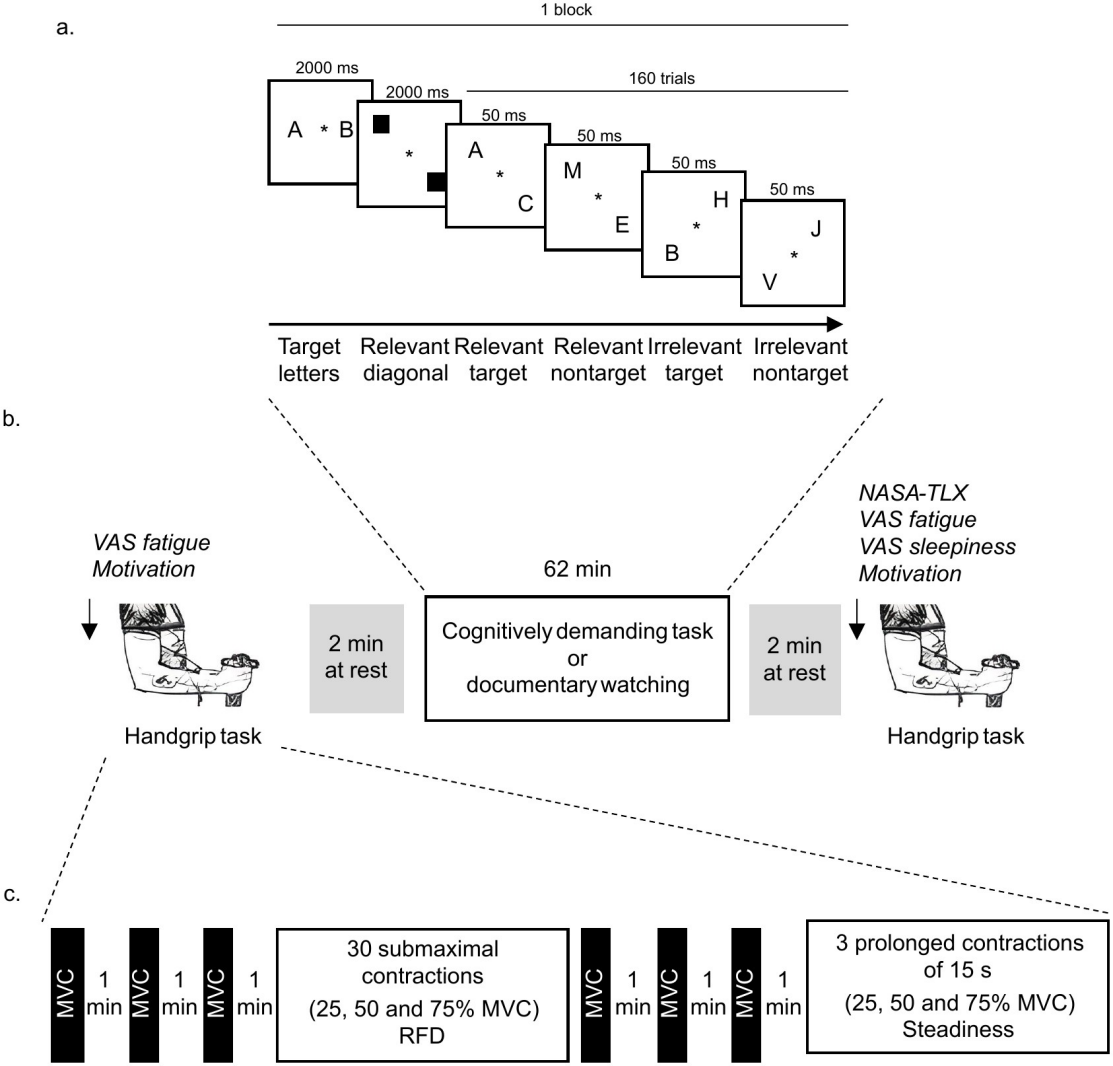
Description of the cognitively demanding task (a), overview of the experimental protocol (b), and description of the handgrip task (c). VAS = visual analog scale, MVC = maximal voluntary contraction, NASA-TLX = National Aeronautics and Space Administration Task Load Index, RFD = rate of force development.

The second and third visits were randomized and counterbalanced. An overview of each visit is presented in Fig 1. Each visit consisted of performing the handgrip task first (Pre), followed by either the cognitively demanding task or the documentary watching for 62 min. At the session end, participants again performed the handgrip task (Post). In each visit, participants began by reporting their level of mental fatigue and indicating their motivation to perform the handgrip task Pre. After the handgrip task Pre, they indicated their motivation to complete the cognitively demanding task or watch the documentary. Then, participants performed either 62 min of the cognitively demanding task or watched a documentary. After the cognitively demanding task and documentary watching, participants reported their perceived workload to achieve both tasks, as well as their subjective feeling of mental fatigue and sleepiness. Finally, after reporting their motivation, they performed the handgrip task Post. Electroencephalographic (EEG) signals were continuously recorded during the cognitively demanding task and documentary watching, and during a rest period of 2 min before and after these tasks. EMG activity of the flexor digitorum superficialis and profundus muscles was recorded during the handgrip task.

### Psychological measures

#### Motivation

Motivation is defined as “the attribute that moves us to do or not to do something”. Motivation to watch the documentary and to perform both the cognitively demanding task and the handgrip task was measured with the motivation scale developed by Matthews et al. [30]. This questionnaire has seven questions on intrinsic motivation (e.g., “I wish to do my best”) and seven questions on extrinsic motivation (e.g., “I performed this exercise only because of an outside reward”). For each question, there are five possible answers (0 = not at all, 1 = a little bit, 2 = somewhat, 3 = very much, 4 = extremely). The score of each motivation ranges between 0 and 28.

#### Subjective workload

The National Aeronautics and Space Administration Task Load Index (NASA-TLX) was used to evaluate subjective workload [31]. The NASA-TLX is composed of six subscales: mental demand (how much mental and perceptual activity was required?), physical demand (how much physical activity was required?), temporal demand (how much time pressure did you feel due to the rate or pace at which the task occurred?), performance (how much successful do you think you were in accomplishing the goals of the task set by the experimenter?), effort (how hard did you have to work to accomplish your level of performance?), and frustration (how much irritating, annoying did you perceive the task?). The participants had to score each of the items on a scale divided into 20 equal intervals anchored by a bipolar descriptor (e.g., high/low). This score was multiplied by 5, resulting in a final score between 0 and 100 for each subscale.

#### Mental fatigue level

A visual analog scale (VAS) was used to measure subjective feelings of mental fatigue [27] before and after the cognitively demanding task and before and after documentary watching. The VAS consisted of a line measuring 100 mm in length with bipolar end anchors (0 mm = “No tired at all”; 100 mm = “Extremely tired”). The following question was asked to assess the mental fatigue level: “What is your feeling of mental fatigue right now?”. Participants were asked to place a mark along the line to indicate how they currently felt. VAS score is defined by the distance between the first anchor (0 mm: “Not tired at all”) and the mark placed by the participant.

#### Sleepiness level

Another VAS was used to measure feelings of sleepiness [32] after the cognitively demanding task and documentary watching. The VAS consisted of a line measuring 100 mm in length with bipolar end anchors (0 mm = “No sleepy at all”; 100 mm = “Extremely sleepy”). The following question was asked to assess the sleepiness level: “What is your feeling of sleepiness right now?”. Participants were asked to place a mark along the line to indicate how they currently felt. VAS score is defined by the distance between the first anchor (0 mm: “Not sleepy at all”) and the mark placed by the participant.

### Data acquisition and analyses

#### Force data

Handgrip force developed during the contractions was measured by a pressure captor (hand dynamometer TSD121C) connected to the Biopac system MP150 (Systems Inc., Santa Barbara, CA). The transducer was connected to an amplifier DA 100C (Biopac MP 150, System Inc., Santa Barbara, CA), whose output was directed to the AcqKnowledge software (Version 4.1, System CA). The sampling frequency of the force signal was digitized at 2000 Hz. The onset of force production was defined as when the force exceeded the baseline by 2 SD. The baseline is calculated on a 1-s sliding window during the rest period preceding each contraction. The offset of force production was defined as the point when the force became lower than the baseline +2 SD. A visual inspection was performed to adjust the onset of force production when necessary to avoid any error. To analyze MVC, only the best of the three contractions performed at the start and the end of physical exercise was considered.

RFD was calculated for each submaximal contraction as the average slope of the force-time curve (force/time) over the time interval 0 to the peak force. For each handgrip task, linear regression parameters (slope, y-intercept, and coefficient of determination: R^2^) were calculated for each individual. Outliers were removed using the Cook distance methodology. The RFD values at Pre were normalized with respect to the MVC at Pre, and RFD values at Post were normalized with respect to the MVC at Post.

Force steadiness, which represents the variability in force production during intermittent contractions, was calculated as the force coefficient of variation (CV). For each prolonged isometric contraction (i.e., from the onset to the offset of muscle force signal), a time window of 10 s was used to calculate the force steadiness. These 10 s were defined as 5 s before and after the middle of the contraction.

#### EMG data

A recording of EMG activity was taken using circular surface electrodes made of bipolar silver chloride with a recording diameter of 10 mm (Swaromed, Nessler Medizintechnik, ref 1066, Innsbruck, Austria) and an interelectrode distance of 20 mm. To achieve low resistance between the electrodes, the skin was shaved and cleaned with alcohol. The flexor digitorum superficialis (FDS) and profundus (FDP) muscles were chosen to represent handgrip muscle activity, and the electrodes were positioned in the direction of the muscle fibers, in accordance with SENIAM recommendations. The reference electrode was placed on the ipsilateral olecranon. The myoelectrical signal was amplified and digitized using the Biopac MP150 system (Biopac Systems, Inc., Goleta, CA, USA) within a bandwidth frequency ranging from 10 to 500 Hz (common mode rejection ratio = 110 dB, Z Input = 1000 MΩ, gain = 500). EMG signals were recorded at a sampling frequency of 2 kHz. The EMG signals were filtered offline with Matlab to reduce power grid noise. The Root Mean Square (RMS) was calculated for each contraction from the onset to the offset of the EMG burst, with a time constant of 40 ms employed for obtaining successive averaged RMS values in assessing EMG activity during handgrip contraction.

#### EEG data

The electroencephalogram was recorded at 64-scalp sites using a 10–20 system (BioSemi ActiveTwo). Electrodes were placed on the outer left and right canthi to monitor horizontal eye movements, and under the left eye to monitor vertical eye movements. Two additional electrodes were placed on the left and right mastoids. During recording, the reference electrode was the BioSemi ActiveTwo system’s common-mode sense electrode. Signa gel was used to fill each electrode to improve signal transduction. Electrophysiological signals were digitized at a 2048 Hz sampling rate and acquired with ActiView software. The EEG signals were analyzed offline using the EEGLAB toolbox for Matlab (version 14.1). Data were re-referenced against the mean of electrodes A1 and A2, and downsampled to 512 Hz. EEGLAB’s runica routine was used to perform independent component analyses to correct eye movement artifacts. Noisy electrodes were identified using the probability method in EEGLAB (Z = 5) and interpolated with a spherical method when necessary.

During the cognitively demanding task, only trials with correct responses were considered for Event-Related Potential (ERP) analyses. Epochs were averaged separately for each condition and each participant. ERPs were obtained by computing the mean amplitude in the time window for each ERP component and by grand-averaging data across participants. ERPs of interest were defined following the study of Boksem et al. [4]. The P1 was quantified as the average amplitude in the 100–160 ms latency interval and analyzed on the electrodes O1 and O2, and the N1 amplitude was quantified as the average amplitude in the 160–220 ms latency interval and analyzed on the electrodes P7 and P8.

Spectral analysis of the EEG was performed with Fast Fourier Transform (FFT), using the *spectopo* function of the EEGLAB toolbox. EEG power was divided into five frequency bands: delta, 1–4 Hz; theta, 4–7 Hz; alpha, 8–12 Hz; beta, 13–30 Hz; and gamma, 30–40 Hz. Three regions of interest (ROIs) were constituted to perform analyses on spectral data: Frontal (mean of AFz, AF3, AF4, Fz, F1, F2, F3, F4, F5, F6), Central (mean of FCz, FC1, FC2, FC3, FC4, FC5, FC6, Cz, C1, C2, C3, C4, C5, C6, CPz, CP1, CP2, CP3, CP4, CP5, CP6), Parietal (mean of Pz, P1, P2, P3, P4, P5, P6, POz, PO3, PO4, Oz, O1, O2).

### Statistics

All data are presented as means ± standard errors of the mean. Greenhouse-Geisser’s correction to the degrees of freedom was applied when violations of sphericity were present (corrected *p*-values are reported). Only significant effects are reported, except when nonsignificance is relevant for the evaluated hypotheses.

Paired *t*-tests were used to assess the effect of condition (Documentary watching, Mental Fatigue) on sleep duration, motivation, NASA-TLX scores, and sleepiness. Two-way repeated-measures 2 × 2 ANOVAs were used to test the effects of condition (Documentary watching, Mental Fatigue) and time (Pre, Post) on the subjective feeling of mental fatigue, RFD-SF, and brain oscillations at rest independently for each frequency band. For brain oscillations during the cognitively demanding task, a two-way repeated-measures 2 × 3 ANOVA was used to test the effects of condition (Documentary watching, Mental Fatigue) and time on task (Part 1, Part 2, Part 3). The effects on MVC were tested using a three-way repeated-measures 2 × 2 × 2 ANOVA, including the within-subject factors of condition (Documentary watching, Mental Fatigue), time (Pre, Post), and physical fatigue (Start, End). For force steadiness, three-way repeated-measures 2 × 2 × 3 ANOVAs including the within-subject factors of condition (Documentary watching, Mental Fatigue), time (Pre, Post), and intensity (25%, 50%, 75%) were used. Finally, three-way repeated-measures 2 × 2 × 3 ANOVAs including the within-subject factors of diagonal (Relevant, Irrelevant), letter (Target, Nontarget), and time on task (Part 1, Part 2, Part 3) were performed for RTs, miss trials, errors, and ERPs during the cognitively demanding task.

All analyses were performed using the Statistical Package for Social Sciences, version 24 for Windows (SPSS Inc., Chicago, IL, USA). Significant main effects of time and significant interactions were followed up with contrast tests with Bonferroni correction, and only significant results were reported with adjusted *p* values. For each ANOVA, partial eta squared was calculated. Thresholds for small, moderate, and large effects were set at 0.01, 0.07, and 0.14, respectively [33]. Cohen’s *d* was calculated for each *t*-test using JASP (Version 0.13.1.0) [Windows software]. Thresholds for small, moderate, and large effects were set at 0.2, 0.5, and 0.8, respectively [33].

## Results

### Subjective evaluations

The sleep duration for the night preceding the experiment did not differ between groups (*t*_13_ = -.267, *p* = .793, *d* = -.071; Mental Fatigue: 457.1 ± 16.7 min, Documentary watching: 451.8 ± 15.9 min).

**Fig 2:**
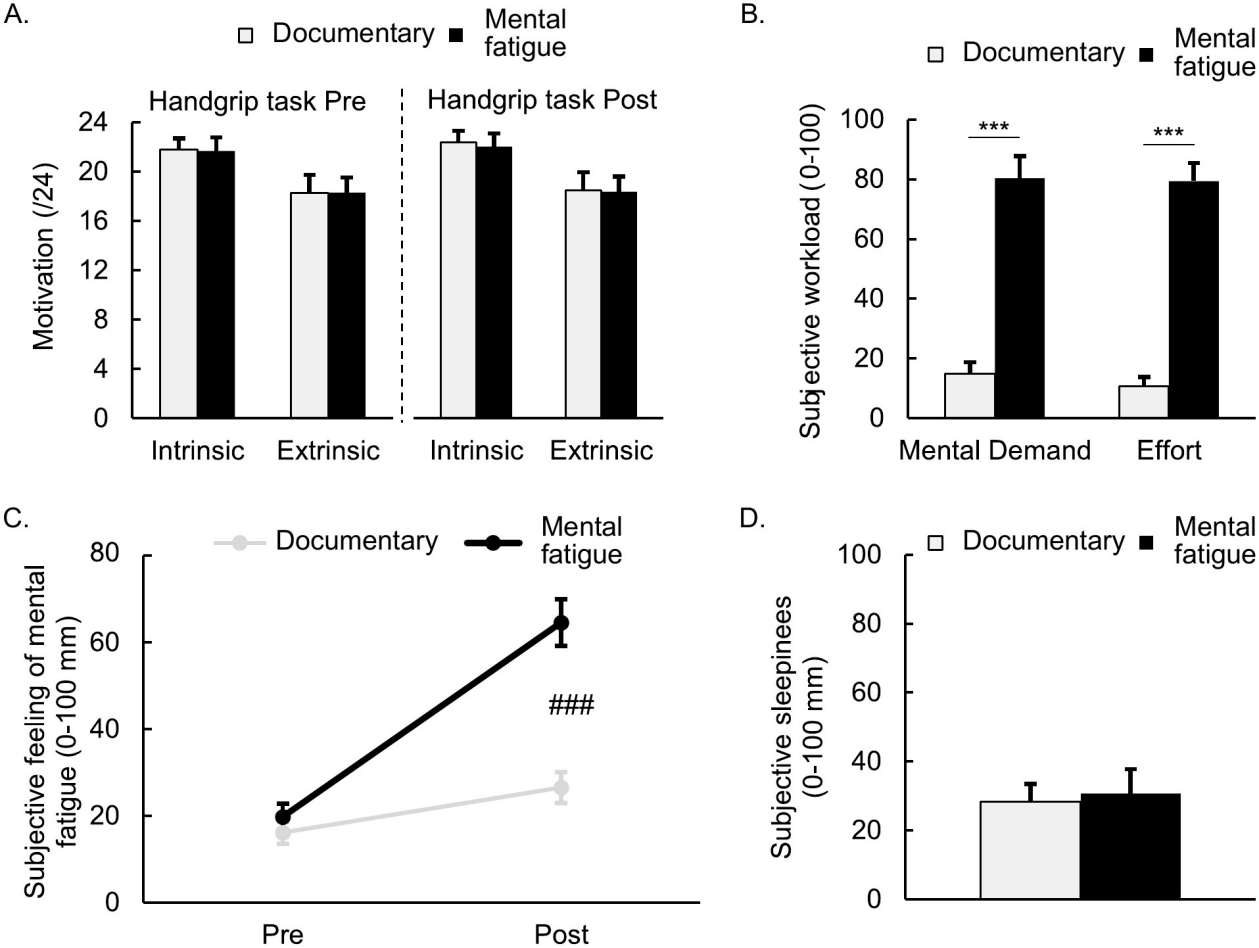
Motivation (A), subjective workload (B), feeling of mental fatigue (C), and sleepiness (D) for the cognitively demanding task and documentary watching. ***: Significant difference between conditions (*p* < .001). ###: Significant interaction effect (*p* < .001). Data are presented as means ± SE (*N* = 14).

No significant differences in motivation were observed to perform the handgrip task at the beginning of the session, either for extrinsic (*t*_13_ = 0.000, *p* = 1.000, *d* = .000; Mental Fatigue: 18.3 ± 1.2, Documentary watching: 18.3 ± 1.5) or intrinsic motivation (*t*_13_ = .144, *p* = .888, *d* = .038; Mental Fatigue: 21.7 ± 1.0; Documentary watching: 21.8 ± 1.0). The motivation did not differ for performing the cognitively demanding task and documentary watching either for extrinsic (*t*_13_ = -1.764, *p* = .101, *d* = -.471; Mental Fatigue: 18.6 ± 1.4; Documentary watching: 15.9 ± 1.5) or intrinsic motivation (*t*_13_ = 0.380, *p* = .909, *d* = .243; Mental Fatigue: 21.9 ± 1.1; Documentary watching: 22.8 ± 0.5, Fig 2A). Finally, no significant differences in motivation were observed to perform the handgrip task at the end of the session either for extrinsic (*t*_13_ = .314, *p* = .759, *d* = .084; Mental Fatigue: 18.4 ± 1.5; Documentary watching: 18.5 ± 1.5) or intrinsic motivation (*t*_13_ = .836, *p* = .418, *d* = .223; Mental Fatigue: 21.9 ± 1.0; Documentary watching: 22.3 ± 1.4, Fig 2A).

Analyses performed on the NASA-TLX revealed that the cognitively demanding task was perceived as more mentally demanding (*t*_13_ = -11.898, *p* < .001, *d* = -3.180, Fig 2B), physically demanding (*t*_13_ = -3.994, *p* = .002, *d* = -1.068), temporally demanding (*t*_13_ = -14.369, *p* < .001, *d* = -3.840), effortful (*t*_13_ = -21.549, *p* < .001, *d* = -5.759, Fig 2), and frustrating (*t*_13_ = -13.226, *p* < .001, *d* = -3.535) than documentary watching.

A significant condition × time interaction was observed for the subjective feeling of mental fatigue (*F*_1, 13_ = 79.858, *p* < .001, $\eta_{p}^{2}=.860$). While the subjective feeling of mental fatigue was similar before both the cognitively demanding task and documentary watching (*t*_13_ = -1.319, *p* = .840, *d* = -.353), a greater increase was reported after the cognitively demanding task (*t*_13_ = -10.351, *p* < .001, *d* = -2.767; 19.7 ± 3.1 to 64.6 ± 5.4), compared to documentary watching (*t*_13_ = -3.165, *p* = .028, *d* = -.846; 16.1 ± 2.6 to 26.5 ± 3.6). The subjective feeling of mental fatigue was higher after the cognitively demanding task than after documentary watching (*t*_13_ = -9.167, *p* < .001, *d* = -2.450; Mental Fatigue: 64.6 ± 5.4, Documentary watching: 26.5 ± 3.6, Fig 2C).

The subjective feeling of sleepiness did not differ after the cognitively demanding task and documentary watching (*t*_13_ = -.353, *p* = .730, *d* = -.094; Mental Fatigue: 30.7 ± 7.0; Documentary watching: 28.4 ± 5.1, Fig 2D).

### Performances during the cognitively demanding task

Reaction times were longer for the relevant (533.2 ms ± 6.3) compared to irrelevant diagonal (425.1 ms ± 5.3; *F*_1, 13_ = 289.515, *p* < .001, $\eta_{p}^{2}=.957$), and for target letters (486.8 ms ± 8.5) compared to nontarget letters (471.5 ms ± 8.0; *F*_1, 13_ = 15.551, *p* = .002, $\eta_{p}^{2}=.545$). A significant time-on-task effect (*F*_2, 26_ = 5.118, *p* = .0013, $\eta_{p}^{2}=.282$) indicated a decrease in reaction times between the first (486.8 ms ± 10.4) and the last part (473.4 ms ± 10.3) of the cognitively demanding task (*t*_13_ = 3.110, *p* = .014, *d* = .831).

Miss trials were higher for the irrelevant (2.8% ± 0.3) compared to the relevant diagonal (2.2% ± 0.3; *F*_1, 13_ = 8.200, *p* = .013, $\eta_{p}^{2}=.387$). A time-on-task effect (*F*_2, 26_ = 7.822, *p* = .011, $\eta_{p}^{2}=.376$) revealed an increase in miss trials between the first (1.5% ± 0.2) and the last part (3.7% ± 0.5) of the cognitively demanding task (*t*_13_ = -3.892, *p* = .002, *d* = -1.040). Errors were also higher for the relevant (14.7% ± 1.4) compared to the irrelevant diagonal (1.4% ± 0.3; *F*_1, 13_ = 78.158, *p* < .001, $\eta_{p}^{2}=.857$) and increased with time-on-task (*F*_2, 26_ = 9.766, *p* = .006, $\eta_{p}^{2}=.429$) with more errors in the last part compared to the first (10.3% ± 1.9 vs. 6.3% ± 1.1; *t*_13_ = -4.361, *p <* .001, *d* = -1.165), and the second part (10.3% ± 1.9 vs. 7.7% ± 1.4; *t*_13_ = -2.802, *p* = .028, *d* = -0.749).

### EEG data

#### ERPs during the cognitively demanding task

**Fig 3:**
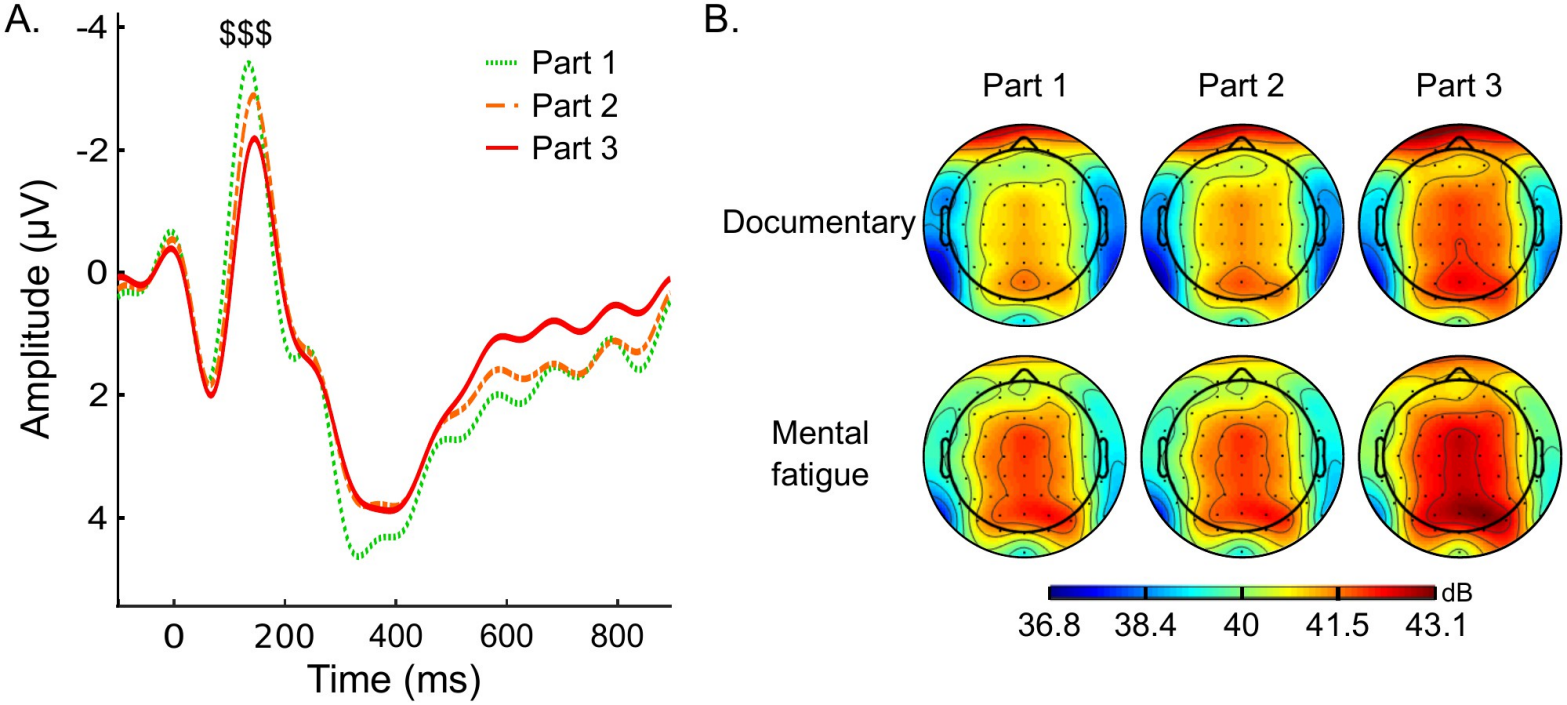
Time course of (A) ERP amplitude at the P8 electrode during the visual attention task and (B) alpha power during both the documentary watching condition (documentary watching) and the mental fatigue condition (visual attention task). $$$: Significant time-on-task effect (*p* < .001).

P1 amplitude showed a larger positive deflection for stimuli presented on the relevant diagonal, compared to stimuli presented on the irrelevant diagonal for both O1 (*F*_1, 13_ = 16.872, *p* = .001, $\eta_{p}^{2}=.565$) and O2 electrodes (*F*_1, 13_ = 10.324, *p* = .001, $\eta_{p}^{2}=.443$). Amplitude was similar for target and nontarget letters. No change in P1 amplitude with time on task could be observed. For N1, analyses showed a significant time-on-task effect on P7 (*F*_2, 26_ = 5.117, *p* = .013, $\eta_{p}^{2}=.282$) and P8 electrodes (*F*_2.26_ = 18.566, *p* < .001, $\eta_{p}^{2}=.588$; Fig 3A), indicating a decrease in N1 amplitude between the first and the last part of the cognitively demanding task (P7: *t*_13_ = -3.198, *p* = .011, *d* = -.855; P8: t_13_ = -6.074, *p* < .001, *d* = -1.623), but also between the first and the second part (t_13_ = -3.460, *p* = .006, *d* = -0.925), and the second and the last part (*t*_13_ = -2.614, *p* = .044, *d* = -.699) for P8 electrode. In addition, a diagonal × letter interaction on P7 (*F*_1, 13_ = 5.469, *p* = .036, $\eta_{p}^{2}=.296$) and P8 electrodes (*F*_1, 13_ = 8.850, *p* = .011, $\eta_{p}^{2}=.405$) indicated that N1 was more pronounced for the target letters in relevant diagonal compared to the nontarget letters in irrelevant diagonal.

#### Brain oscillations during the documentary watching and mental fatigue

Analyses showed a significant time-on-task effect on the parietal region (*F*_2, 26_ = 6.319, *p* = .017, $\eta_{p}^{2}=.327$) with an increase in theta power between part 1 and part 3 (*t*_13_ = -3.424, *p* = .006, *d* = -0.125; + 0.5%). In addition, a time-on-task × condition interaction in frontal (*F*_2, 26_ = 4.828, *p* = .017, $\eta_{p}^{2}=.271$), and central regions (*F*_2, 26_ = 7.204, *p* = .003, $\eta_{p}^{2}=.357$) indicated an increase in theta power between part 1 and part 3 only during the documentary watching (frontal: *t*_13_ = -4.183, *p* = .004, *d* = -1.118; + 1.0%; central: *t*_13_ = -3.228, *p* = .026, *d* = -0.863; + 1.0%).

For alpha power, analyses revealed a significant condition effect for frontal (*F*_1, 13_ = 6.940, *p* = .021, $\eta_{p}^{2}=.348$), central (*F*_1, 13_ = 14.031, *p* = .002, $\eta_{p}^{2}=.519$), and parietal regions (*F*_1, 13_ = 6.538, *p* = .024, $\eta_{p}^{2}=.335$) reflecting a greater alpha power during the cognitively demanding task compared to the documentary watching. A time effect on frontal (*F*_2, 26_ = 17.844, *p* < .001, $\eta_{p}^{2}=.578$), central (*F*_2, 26_ = 18.930, *p* < .001, $\eta_{p}^{2}=.593$), and parietal regions (*F*_2, 26_ = 20.034, *p* < .001, $\eta_{p}^{2}=.606$) evidenced an increase in alpha power between part 1 and part 2 (frontal: *t*_13_ = -2.705, *p* = .036, *d* = -0.085, *d* = -1.118; + 1.0%; central: *t*_13_ = -2.719, *p* = .034, *d* = -0.091), part 2 and part 3 (frontal: *t*_13_ = -3.260, *p* = .004, *d* = -0.102; central: *t*_13_ = -3.421, *p* = .006, *d* = -0.114; parietal: *t*_13_ = -3.737, *p* = .003, *d* = -0.136), and between part 1 and part 3 (frontal: *t*_13_ = -5.965, *p <* .001, *d* = -0.187; central: *t*_13_ = -6.140, *p <* .001, *d* = -0.205; parietal: *t*_13_ = -6.293, *p <* .001, *d* = -0.229).

#### Brain oscillations at rest

Analyses revealed a significant condition effect on the frontal region (*F*_1, 13_ = 4.943, *p* = .044, $\eta_{p}^{2}=.275$), indicating a greater alpha power for the cognitively demanding task compared to the documentary watching. No significant effects were reported for theta power.

### Physical performances

#### Maximal voluntary contraction

Analyses showed a significant time effect (*F*_1, 13_ = 5.182, *p* = .040, $\eta_{p}^{2}=.285$), evidencing a significant decrease in maximal force production (- 2.8%) between pre (167.5 N ± 7.2) and post measurements (162.8 N ± 6.8; Fig 5A).

#### Rate of force development

**Fig 4:**
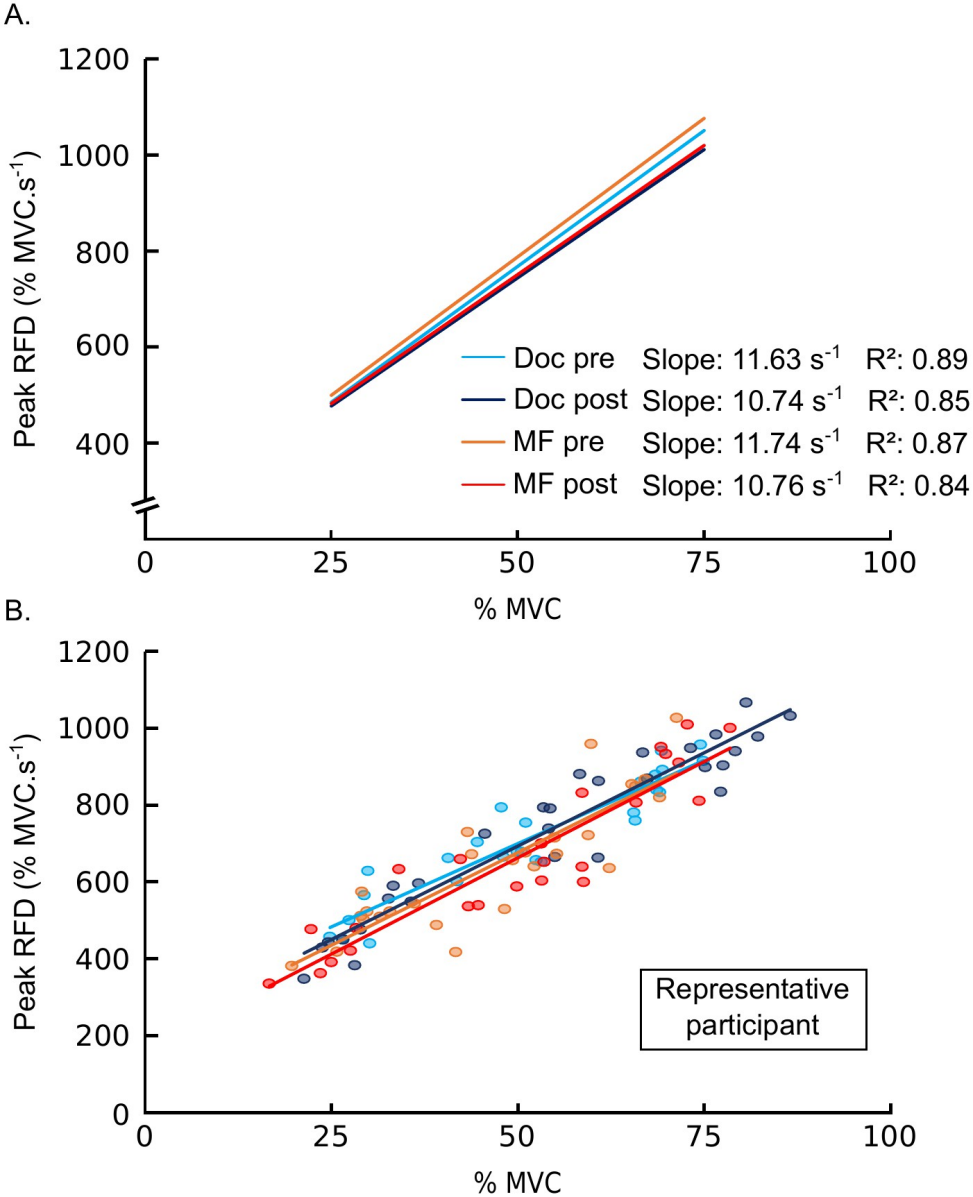
RFD-SF over all participants (A), and for one representative participant (B).

For the RFD, a significant effect of time was observed on the slope (*F*_1, 13_ = 6.443, *p* = .025, $\eta_{p}^{2}=.331$), indicating a decrease in slope (Pre = 11.63 ± 0.87; Post = 10.75 ± 0.91; see Fig 4). In addition, a significant effect of time was also observed for *R*^*2*^ (*F*_1, 13_ = 8.882, *p* = .011, $\eta_{p}^{2}=.406$), evidencing a greater dispersion of data after the completion of both the cognitively demanding task and documentary watching (Pre = 0.88 ± 0.01; Post = 0.84 ± 0.02). However, no significant effects were observed for the intercept. In addition, no significant effects were reported for RMS EMG activity, either for the FDS or the FDP muscles (all *p*-values > .05).

#### Force steadiness

**Fig 5:**
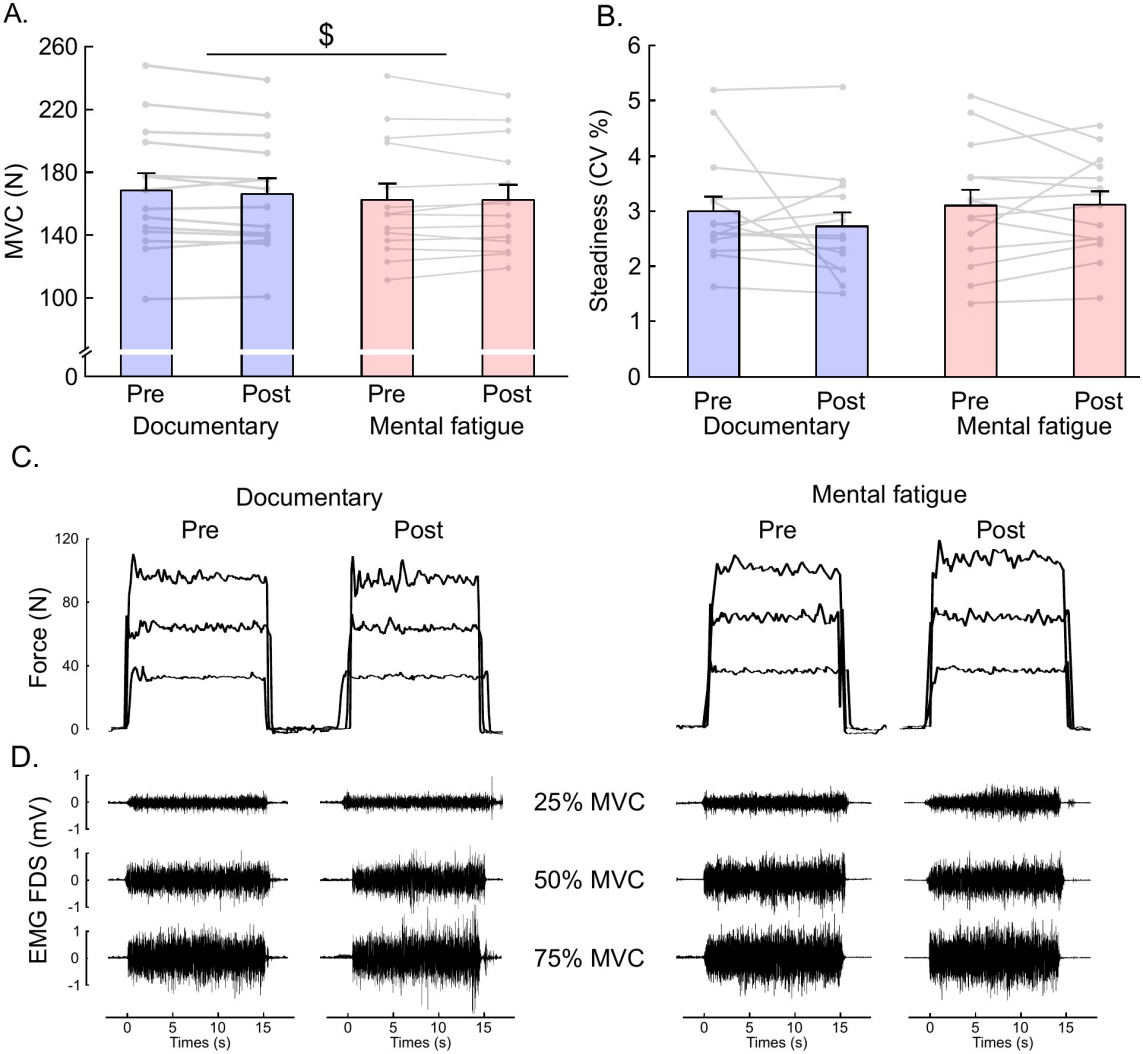
Effects of documentary watching and mental fatigue on maximal force production (A), and force steadiness (B). Illustration of force output at 25%, 50%, and 75% of MVC (C), and surface EMG activity for the FDS (flexor digitorum superficialis) muscle (D) for one individual participant. Data are presented as mean ± SEM (*N* = 14), and light grey bars indicate individual performance. $: Significant time-on-task effect (*p* < .05).

Analyses performed on force steadiness during the submaximal isometric contractions did not show any difference on CV (all *p*-values > .10, $\eta_{p}^{2}<.14$; Mental Fatigue: 2.88% ± 0.1; Documentary watching: 2.94% ± 0.2; see Fig 5B). A significant intensity effect was reported for the EMG activity of the FDS muscle (*F*_2, 26_ = 177.693, *p* < .001, $\eta_{p}^{2}=.932$), indicating that EMG activity was higher for contractions at 50% (55% of RMS_max_ ± 1.5) of MVC compared to 25% of MVC (28% of RMS_max_ ± 1.1; *t*_13_ = -8.843, *p* < .001, *d* = -2.363), and for contractions at 75% of MVC (89% of RMS_max_ ± 2.4) compared to 25% of MVC (*t*_13_ = -18.840, *p* < .001, *d* = -5.035; 28% of RMS_max_ ± 1.1) and 50% of MVC (*t*_13_ = -9.997, *p <* .001, *d* = -2.672; 55% of RMS_max_ ± 1.5).

## Discussion

The present study aimed to investigate the effects of mental fatigue on force production capacities. To induce mental fatigue, participants engaged in a 62-minute visual attention task, which effectively led to increased subjective feelings of mental fatigue and a decline in behavioral performance, characterized by an increase in missed trials and errors, and a reduction in N1 amplitude. Regarding force production capacities, the results revealed a significant decrease in maximal force production and an impaired ability to perform quick contractions at submaximal intensities after both the cognitively demanding and documentary-watching tasks. However, it is important to note that these effects were observed in both conditions and therefore, cannot be solely attributed to mental fatigue.

The present study utilized a 62-minute visual attention task to induce mental fatigue, a method previously successful in inducing mental fatigue for three hours in another research by Boksem et al. [4]. Our participants reported a significant increase in subjective mental fatigue for both the visual attention task and the documentary watching, but the increase was more pronounced in the visual attention task. Notably, participants also reported higher mental and physical demand, temporal demand, effort, and frustration during the visual attention task compared to the documentary watching, providing subjective evidence of induced mental fatigue. Additionally, electrophysiological measurements, specifically changes in brain oscillations, supported the presence of mental fatigue. A meta-analysis had previously highlighted consistent changes in theta and alpha power as markers of mental fatigue [28]. Furthermore, prolonged engagement in demanding cognitive tasks increases both theta [34] and alpha [35] power. In the present study, a significant increase in theta and alpha power was observed during both the visual attention task and documentary watching. However, this increase in both theta and alpha power could reflect different cognitive processes. Indeed, the increase in theta power during the visual attention task could be linked with increased cognitive load [36, 37], while the increase during the documentary watching could reflect sleepiness [38]. The rise in alpha power may reflect mind wandering during documentary watching [39], while it can be interpreted as an engagement of cognitive resources during the visual attention task [40]. Due to the concomitant changes in brain oscillations in both mental fatigue and documentary watching, they cannot be explicitly attributed to an increase in mental fatigue.

Watching a documentary is commonly used as a control condition in mental fatigue experiments [16, 24] and has recently been suggested as a good control task [29]. However, in this study, watching a documentary induced drowsiness, which seemed to lead to a decline in force production capacities similar to that observed after mental fatigue induced by a cognitively demanding task. In this context, the use of a single documentary for all participants may not be the most optimal control task. Instead, consideration could be given to presenting several emotionally neutral documentaries and allowing participants to choose topics of interest to them, thereby promoting greater engagement and reducing the likelihood of drowsiness onset.

Despite inducing mental fatigue through the 62-minute visual attention task, we did not observe any increase in RTs over time during the cognitively demanding task; in contrast, participants responded increasingly quickly. This unexpected finding may be attributed to learning effects, as evidenced by improved RTs, which may have outweighed the impact of mental fatigue within the given period. The relatively shorter duration of the task compared to a previous study [4], where significant RT increases were observed during a 3-hour cognitive task, further supports the possibility of learning effects mitigating the effects of mental fatigue.

Although many studies reported an alteration of performance during a prolonged cognitively demanding task [4, 41], other studies evidenced maintenance or improvement of performances, which is probably linked with learning effects and/or an important motivation [42]. In our study, despite improved RTs, an increase in missed trials was observed, similar to the findings of Boksem et al. [4]. This combination suggests that participants remained highly engaged in the task, but it became increasingly challenging to identify target stimuli correctly. Thus, the performance deterioration observed in the presence of mental fatigue is more likely attributed to the difficulty in target identification rather than task disengagement.

Additionally, our study focused on two early visual components, the P1 and N1 components, in event-related potential (ERP) analyses. The P1 component, indicative of selective spatial attention, exhibited a smaller amplitude for the irrelevant diagonal, consistent with existing literature on focused attention [43]. Notably, mental fatigue did not affect early sensory processing and selective spatial attention, as the absence of time-on-task effect on P1 amplitude suggests. Regarding the N1 component, which reflects visual discrimination processes, a larger amplitude was observed for attended stimuli (relevant diagonal) compared to nonattended stimuli (irrelevant diagonal). This aligns with previous findings indicating increased N1 amplitude for stimuli presented in an attended position [44]. However, a significant decrease in N1 amplitude over time was observed, similar to the results obtained by Boksem et al. [4]. This reduction could indicate a decrease in top-down modulation of sensory processing in the presence of mental fatigue [41], or be attributed to an increase in mental workload during the cognitively demanding task. In summary, our ERP analyses provided evidence of the presence of mental fatigue, either through an increase in mental workload over time and/or a decrease in attention during the completion of the cognitively demanding task.

In the present study, we evidenced decreased hand MVC force (≈ 2.8%) after the cognitively demanding task. At first sight, this observation could be surprising because some studies on mental fatigue suggest that a prolonged cognitive task does not induce a reduction of maximal force production [1, 3]. However, other studies on mental fatigue have already reported a decrease in MVC. Budini et al. [6] reported a significant reduction in maximal force production of knee extensors after completing 100 min of a cognitively demanding task. The authors explained this result by demotivation in repeating an MVC task after completing the mental fatigue protocol. It has also been shown that individuals described as “not motivated” could not generate a sufficient central command during a knee extension task with a resultant force decline [45]. Contrary to Budini et al. [6], our participants’ motivation was checked before and after both the cognitively demanding task and documentary watching. No significant difference was reported, ruling out the interpretation that the loss of maximal strength is related to a decrease in motivation.

Another explanation for this result could be an insufficient warm-up before performing the MVC after both the cognitively demanding task and documentary watching. Mangin et al. [29] have recently reported a decrease in MVC during handgrip contractions after both a mentally fatiguing task (Stroop task of 30 min) and documentary watching in proportions similar to those observed in our study (≈ 3.6%). An absence of warm-up prior to the completion of the handgrip contractions could impair some peripheral parameters of neuromuscular function [46]. It has been previously suggested that slightly increasing muscle fibers recruitment could compensate for an absence of warm-up to preserve knee extensors MVC [18], but it may not be valid for handgrip contractions. In addition to the potential explanations provided for the observed decrease in hand MVC force (≈ 2.8%) following the cognitively demanding task, it is crucial to consider the calculation of the minimal detectable change (MDC) [47, 48]. With a threshold set for detectable changes at 4.9% of MVC, a value higher than the reported decrement of 2.8% in MVC force, it becomes imperative to approach this decline with caution. Despite the statistical significance, the MDC prompts careful consideration of this result, shedding light on why findings on the effects of mental fatigue often vary. The absence of MDC reporting in most studies underscores the importance of including this parameter to enhance clarity regarding the effects of mental fatigue on maximal force production capacity.

Finally, another explanation for the decrease in maximal strength could be the appearance of sleepiness after both tasks. Indeed, studies reported that sleepiness could induce a decrease in maximum knee extensions [49] and handgrip strength following sleep deprivation protocols [50]. It would be interesting to follow the evolution of sleepiness during the experiment more closely to control for this confounding factor and to investigate, more specifically, the effects of mental fatigue. Using complex dual tasks for a short duration rather than continuous single cognitive tasks for an extended period to induce mental fatigue could be more appropriate to study the phenomenon of mental fatigue per se by reducing the apparition of sleepiness [51].

When an isometric contraction is performed, force fluctuations can be observed. When force fluctuations increase, force steadiness decreases, and vice versa. An increase in force fluctuations has been observed with an increase in contraction intensity for both the upper [52] and lower limbs [53]. However, another study reported that during short contractions (i.e., 6 s), force fluctuations did not become higher in contractions at 80% MVC compared to contractions at 30% MVC [54]. The short contraction duration (i.e., 3s) in the present study could explain why the intensity of the contraction did not impact the force steadiness.

In the present study, mental fatigue did not impact force steadiness during submaximal isometric handgrip contractions. Our result is consistent with a recent study, which did not reveal any effects of 100 min of cognitively demanding task on force steadiness during isometric pinch contractions [24], but also with another study performed on lower limb evidencing the maintenance of force steadiness during submaximal isometric knee extensions after 50 min of motor imagery [16]. A mental effort could impact force steadiness but only when mental fatigue is induced during muscle contractions with a concomitant cognitively demanding task [55]. The effects could be attributed to cognitivomotor interference [56] due to the simultaneous execution of two different tasks, as proposed by Budini et al. [24].

RFD is more sensitive to changes induced by neuromuscular training than MVC force, and it is considered as a critical component of sports performance and daily life activities [9]. A previous study showed that mental fatigue did not influence RFD during submaximal knee extensions at 50% of MVC [16]. However, because most life activities are performed at submaximal intensities, we chose to focus on the effects of mental fatigue on RFD-SF. Firstly, it is important to note that the RFD-SF assessment protocol did not induce significant muscle fatigue (e.g., a decrease in MVC); thus, there is no confounding factor. The present study evidences an impairment in the slope of the RFD-SF after both the cognitively demanding task and the documentary watching. While studies suggested that RFD is linked with both neural (e.g., motor unit recruitment and discharge rate) and muscular determinants (e.g., microfibrillar mechanisms, muscle size, and architecture) [9], Boccia et al. [11] suggested that peripheral neuromuscular properties did not affect the RFD-SF. A study conducted in individuals with multiple sclerosis proposed that RFD-SF modulations were due to deficiencies in the central nervous system (e.g., reduced neural drive and maximal motor unit discharge rates). Although mental fatigue did not seem to impair neural drive to the muscle [57], a recent study evidenced a decrease in motor unit firing rate after a mentally fatiguing task for dorsiflexion contractions at 20% of MVC for males and females, and at 50% of MVC for males [58]. The decrease in anterior cingulate cortex (ACC) activity, a brain region connected with motor planning regions such as the prefrontal cortex and supplementary motor areas, in the presence of mental fatigue [59] could induce changes in the activation of synergistic muscle groups or changes in the co-contraction [58] and could explain the decrease in motor unit firing rate. A previous study also evidenced a reduction in motor unit firing rate during isometric plantarflexion at 20% of MVC [19] after a mentally fatiguing task and a documentary watching. Therefore, a reduction in motor unit firing rate could also be associated with the impairment of RFD-SF observed in our two experimental conditions. In addition, the present study also evidenced an impairment in *R*^*2*^ (coefficient of determination) that reveals the consistency with which the central nervous system regulates the RFD across force levels [15]. The origin of *R*^*2*^ is attributed to modulation in agonist-antagonist coordination during muscle contraction [13]. Therefore, mental fatigue may impair neuromuscular quickness through changes in the central nervous system caused by the influence of mental fatigue on ACC activity. Nonetheless, as effects (i.e., impairment in the slope of the RFD-SF in and *R*^*2*^) were reported after both the cognitively demanding task and the documentary watching, further investigations are needed to validate this hypothesis.

The findings from this study have important implications for coaches and sports athletes aiming to optimize performance during training and competition. Both prolonged attentional tasks and passive activities like watching a documentary can decrease maximal force production and impair the ability to perform quick contractions at submaximal intensities. Coaches should be aware that these effects on force production capacities are not specific to mental fatigue alone but can also result from other cognitive activities. Therefore, it is crucial to consider the potential impact of cognitive engagement, mental workload, and motivation when assessing athletes’ performance. Coaches may need to adjust training regimens, providing adequate mental rest and recovery periods to mitigate the negative effects of prolonged cognitive engagement on physical capabilities. Furthermore, it may be beneficial for athletes to incorporate specific warm-up routines before engaging in force-based activities to minimize any potential performance decrements. Developing strategies to manage mental fatigue and maintaining proper focus during physically demanding tasks can be key to maintaining optimal force production capacities and overall sports performance. Finally, understanding the interaction between cognitive demands and physical capabilities can help athletes and coaches design more effective training programs and achieve better results.

## Conclusion

In conclusion, the completion of the prolonged attentional task successfully induced an increase in subjective mental fatigue. During this study, mental fatigue was objectively evaluated by a decrease in behavioral performances (increase in missed trials and errors) and a reduction in N1 amplitude that could be interpreted as an increase in mental workload over time and/or a decrease in attention to the completion of a cognitively demanding task. However, despite a greater alpha power during the cognitively demanding task, brain oscillations did not evidence specific changes associated with mental fatigue. We found that force steadiness remained relatively preserved regarding hand force production capacities. However, our study revealed a significant decrease in MVC force, even if this decrement is lower than the minimal change detectable. Additionally, there was a compromised ability to execute quick contractions at submaximal intensities. Although the effects of mental fatigue on force production capacities were not directly observed, as they occurred after both the mentally fatiguing task and documentary watching, it is possible that mental fatigue and its impact on the anterior cingulate cortex (ACC) activity could influence central-level modulations during muscle contractions, such as motor unit firing rate and agonist-antagonist coordination. These findings highlight the importance of managing mental fatigue in athletes and coaches to optimize sports performance. Further research is needed to elucidate the effects of mental fatigue on force production capacities in both upper and lower limb muscles, particularly the ability to perform quick contractions at submaximal intensities. In addition, studying the impact of mental fatigue on more isolated movements, such as pinching or wrist flexion and extension, would provide valuable insights into the effects on hand force production capacities, albeit less environmentally friendly. Furthermore, an extension of our field of study to other precision hand tasks, such as the Purdue Pegboard Test and the Nine-Hole Peg Test, could be of interest. This broader approach could contribute to a better understanding of the influence of mental fatigue on manual motor skills.

## Supporting information

S1 Table Subjective data. (XLSX)

S2 Table Behavioral and physiological data of cognitive task. (XLSX)

S3 Table Data for brain oscillations. (XLSX)

S4 Table Data for physical tasks. (XLSX)
